# Complete genome sequence of an enterotoxigenic *Bacteroides fragilis* strain 2-078382-3 from an infant with diarrhea

**DOI:** 10.1128/mra.00587-24

**Published:** 2024-12-20

**Authors:** Eunbi Lee, Heekyoung Kang, Hyun Beom Song, Jinki Yeom

**Affiliations:** 1 Department of Biomedical Science, College of Medicine, Seoul National University, Seoul, South Korea; 2 Cancer Research Institute, Seoul National University, Seoul, South Korea; 3 Institute of Endemic Diseases, Seoul National University Medical Research Center, Seoul National University College of Medicine, Seoul, South Korea; 4 Department of Tropical Medicine and Parasitology, Seoul National University College of Medicine, Seoul, South Korea; 5 Department of Microbiology and Immunology, College of Medicine, Seoul National University, Seoul, South Korea; University of Maryland School of Medicine, Baltimore, Maryland, USA

**Keywords:** *Bacteroides fragilis*, toxin, gut inflammation, colorectal cancer

## Abstract

This study presents that *Bacteroides fragilis* strain 2-078382-3, isolated from an infant with diarrhea, has a 5.2 million base pair chromosome with a 43.2% GC content and a 5,600 base pairs plasmid pBFP53 with a 39.6% GC content. The strain contains 4,262 genes, including 4,105 coding sequences.

## ANNOUNCEMENT

The enterotoxigenic *Bacteroides fragilis* (ETBF) strain 2-078382-3 (ATCC 43858), isolated from an infant with diarrhea in Arizona, has been extensively studied to understand ETBF pathogenic mechanisms (1, 2). Known for its high toxin production and virulence (3), this strain’s genome was previously sequenced (whole-genome sequencing [WGS] project LIDV01) but only available at the contig level. This study presents the complete genome sequencing of ETBF strain 2-078382-3 from American Type Culture Collection (ATCC 43858), providing a more comprehensive understanding of its genetic composition and pathogenicity.

For sample preparation, a single colony of ETBF strain 2-078382-3 was grown in 30 mL of Brain Heart Infusion medium (Difco, 237500) with supplementation of yeast extract (5 mg/mL), hemin (5 mg/mL), vitamin K (0.5 mg/mL), gentamicin (200 μg/mL), and L-cysteine (0.5 mg/mL). The strain underwent two passages before sequencing. Culture incubation occurred in an anaerobic chamber (Whitley DG250) using a gas mixture of 10% H_2_, 10% CO_2_, and 80% N_2_ at 37°C for 48 hours. Genomic DNA extraction utilized the QIAamp DNA Mini Kit (Qiagen) following the manufacturer’s instructions, and the isolated DNA underwent both Nanopore and Illumina sequencing.

For Nanopore sequencing, DNA preparation involved end-repair and dA-tailing using NEBNext FFPE DNA Repair Mix (30 µL, New England Biolabs, USA), followed by adapter ligation to DNA fragments (up to 20 kb) using NEBNext Quick Ligation Module to create a Flongle flow cell-compatible library. The library was loaded into the Flongle flow cell on the MinION device (Oxford Nanopore Technologies, UK), with sequencing performed using MinKNOW software (v23.04.5). Raw data (FAST5) conversion to FASTQ format employed Guppy basecaller (v6.5.7, ONT) with a quality threshold of 7.

Illumina sequencing library preparation followed the TruSeq Nano DNA High Throughput Library Prep Kit (Illumina) manufacturer’s instructions. The process began with shearing 100 ng DNA into smaller fragments using adaptive focused acoustic technology (Covaris). The fragmented DNA underwent end-repair to create blunt-ended, 5′ phosphorylated double-stranded DNA molecules. Size selection using a bead-based method achieved a suitable fragment size (150 bp) for Illumina sequencing. Before quality control, we confirmed the quality of Illumina reads using FastQC. FastQC confirmed high-quality reads (above Q30), eliminating the need for additional quality control. The statistical information for Nanopore and Illumina sequencing is shown in Table 1.

**TABLE 1:** Sequencing data and genomic features of enterotoxigenic *Bacteroides fragilis* (ETBF) strain 2-078382-3 and parameters of tools

Sequencing data information			
Sequencing method	Nanopore		Illumina
Total bases (bp)	242,107,409		3,246,538,052
Total reads	5,600		21,500,252
N50 (bp)	13,111		
GC content	42.4%		43.3%
AT content	57.6%		56.7%
Coverage (×)	46.6		625.0
SRA accession no.	SRR28879121		SRR28879120

Genomic information			
Genomic element	Chromosome		pBFP53
Size (bp)	5,188,551		5,594
GC content	43.2%		39.6%
Number of coding sequences	4,169		7
Number of rRNAs	93		0
Number of tRNA	73		0
GenBank accession no.	CP142686		CP142687

The parameter of FLYE (v. 2.9)			
Parameter		Value	
big_genome_threshold		29,000,000	
low_cutoff_warning		1	
hard_min_coverage_rate		10	
assemble_kmer_sample		1	
repeat_graph_kmer_sample		1	
read_align_kmer_sample		1	
maximum_jump		1,500	
maximum_overhang		1,500	
repeat_kmer_rate		100	
assemble_ovlp_relative_divergence		0.10	
repeat_graph_ovlp_divergence		0.15	
read_align_ovlp_divergence		0.25	
max_coverage_drop_rate		5	
chimera_window		100	
min_reads_in_disjointig		4	
max_inner_reads		10	
max_inner_fraction		0.25	
add_unassembled_reads		0	
max_separation		500	
unique_edge_length		50,000	
min_repeat_res_support		0.51	
out_paths_ratio		5	
graph_cov_drop_rate		5	
coverage_estimate_window		100	
extend_contigs_with_repeats		1	
min_read_cov_cutoff		3	
short_tip_length		10,000	
long_tip_length		100,000	
max_bubble_length		50,000	

The parameter of Polypolish (v0.5.0)			
Parameter		Value	
ORIENTATION		Auto	
LOW		0.1	
HIGH		99.9	
FRACTION_INVALID		0.2	
FRACTION_VALID		0.5	
MAX_ERRORS		10	
MIN_DEPTH		5	

Genome assembly employed FLYE (v. 2.9) (4) for Nanopore reads and Polypolish (v0.5.0) for Illumina read polishing (5), using default parameters unless specified (Table 1). The process began with creating a circular draft genome using Nanopore sequencing via FLYE (v. 2.9), followed by accuracy improvement using Illumina reads with Polypolish (v0.5.0). The assembled circular DNA showed no rotation to a specific base. The final genome consists of a 5,188,551 base pair chromosome (43.2% GC) and a 5,594 base pair plasmid pBFP53 (39.6% GC). NCBI PGAP (version 6.4) annotation (6, 7) revealed 4,262 genes and 4,105 coding sequences, with the complete genome sequence available in GenBank (Table 1).

## Data Availability

The data that support the findings of this study are openly available in GenBank of NCBI under accession nos. CP142686 and CP142687 for complete genome sequencing. SRR28879121 for the Nanopore sequencing data and SRR28879120 for the Illumina sequencing data are deposited in the NCBI database.
